# Multiple osteochondromas of the antlers and cranium in a free-ranging white-tailed deer (*Odocoileus virginianus*)

**DOI:** 10.1371/journal.pone.0173775

**Published:** 2017-03-15

**Authors:** Uwe Kierdorf, Karl V. Miller, Stefan Flohr, Santiago Gomez, Horst Kierdorf

**Affiliations:** 1 Department of Biology, University of Hildesheim, Hildesheim, Germany; 2 Warnell School of Forestry and Natural Resources, University of Georgia, Athens, United States of America; 3 Department of Pathological Anatomy, University of Cádiz, Cádiz, Spain; Colorado State University, UNITED STATES

## Abstract

This paper reports a case of multiple osteochondromas affecting the antlers and the left zygomatic bone of a free-ranging adult white-tailed buck (*Odocoileus virginianus*) from Georgia, USA. Along with a few postcranial bones, the antlered cranium of the individual was found in a severely weathered condition and devoid of any soft tissue. The antlers exhibited five pedunculated exostoses that were composed of cancellous bone and, in their peripheral portions, also mineralized cartilage. The largest of the exostoses, located on the right antler, had a maximum circumference of 55 cm. The exostosis arising from the zygomatic bone was broad-based and much smaller than the exophytic outgrowths on the antlers. Diagnosis of the exostoses as osteochondromas was based on their overall morphology, the normal bone structure in their stalk regions, and the continuity of their spongiosa and cortex with the respective components of the parent bones. Antleromas, i.e., pathological outgrowths developing on antlers as a result of insufficient androgen production, were excluded in the differential diagnosis, based on (1) the apparent maturity and, except for the tumors, normal shape of the antlers and (2) the fact that exostosis formation had also affected the zygomatic bone. Previously only a single case of solitary osteochondroma of an antler has been described in the scientific literature. The case presented here is the first report of multiple osteochondromas in a deer. As antlers are regularly collected as trophies, and huge numbers of them are critically inspected each year, the fact that thus far only two cases of antler osteochondromas have been reported suggests that these tumors are very rare.

## Introduction

An osteochondroma (osteocartilaginous exostosis) is a benign osseocartilaginous excrescence that arises from the surface of a bone [1–7]. Osteochondromas can be present as either solitary lesions or in the form of multiple exostoses, a condition also known as osteochondromatosis or multiple cartilaginous exostoses [1–8]. In humans, solitary osteochondromas account for about 35% of all benign bone tumors [6]. Our current understanding of the pathogenesis of osteochondromas is limited, and the cellular origin of osteochondromas is a matter of debate [4, 9]. Osteochondromas vary markedly in size and shape. They can be pedunculated with a narrow stalk and bulbous tip, or broad-based (sessile osteochondromas). Sometimes, the surface of an osteochondroma has a cauliflower-like appearance [1–3, 5]. A radiographically and histologically demonstrable characteristic of osteochondromas is the continuity of their spongiosa (or marrow cavity) and cortex with those of the parent bone from which they developed [3–7]. This diagnostic feature is useful in the differentiation of osteochondromas from other hyperplastic or neoplastic bone masses [3, 5–7].

Osteochondromas are characterized by a cap of hyaline cartilage that can cover the entire surface of sessile tumors, but in pedunculated ones is present only at the tip of the exostosis [3, 5–7]. This cartilage cap, which may be completely reduced following cessation of growth, resembles a growth plate and can show variably large areas of mineralization [3–7]. The more basal portions of an osteochondroma consist of cancellous bone formed by endochondral ossification [3–6]. Islands of mineralized cartilage can be embedded within this cancellous bone that is not remodeled [5–7]. Malignant transformation of an osteochondroma into a chondro- or osteosarcoma is possible [3, 5–7, 10]. While thin, well defined cartilage caps with regular, stippled areas of mineralization are consistent with a benign tumor, thick and poorly defined cartilage caps showing an irregular pattern of mineralization are suggestive of malignant transformation [3, 5, 6]. In humans and other mammals, osteochondromas occur chiefly in bones developing by endochondral ossification [3, 5–7]. Most osteochondromas develop in the metaphyseal regions of long limb bones close to growth plates [1, 3–7]. In humans, predilection sites for osteochondroma formation are the distal metaphysis of the femur and the proximal humeral and tibial metaphyses [3, 5]. Growth of osteochondromas usually takes place in parallel with that of its parent bone and mostly ceases upon epiphyseal closure [3, 6, 7]. Some osteochondromas may even spontaneously regress [6].

Osteochondromas of the skull are rare. Principal locations in the human mandible are the condyle [11] and the coronoid process [12], i.e., sites of cartilage proliferation and endochondral ossification. In the human cranium, osteochondromas are usually present in the region of the cranial base that is likewise formed by endochondral ossification [3, 13]. However, very rarely osteochondromas have also been reported from cranial bones developing by intramembranous ossification [14].

There has been some discussion about whether osteochondromas should be classified as cases of skeletal dysplasia rather than true neoplasms [3, 6, 7]. As both solitary and multiple osteochondromas have been linked to loss-of-function mutations in *EXT1* or *EXT2* genes encoding exostosin-1 and exostosin-2 glycosyltransferase, respectively [5, 6], it was recently concluded that an osteochondroma should be considered a unique form of benign bone tumor rather than a hamartoma [6]. Some investigators regard *EXT1* and *EXT2* to represent tumor suppressor genes [5]. Exostosin-1 and 2 function in the biosynthesis of heparan sulfate (HS), which constitutes the glycosaminoglycan moiety of cell surface and matrix-associated proteoclycans, referred to as heparan sulfate proteoglycans (HSPGs) [9, 15]. HSPGs are involved in numerous signaling pathways and have important functions during development and in the maintenance of cellular functions during adult life [9, 15]. In most cases of solitary and multiple osteochondromas, the cartilage cap contains a mixture of mutated (HS-deficient) and wild-type cells [6].

In the case of solitary osteochondromas, inactivation of *EXT* genes is limited to cells of the cartilage cap [6]. In humans, domestic dogs and horses, osteochondromatosis occurs as a hereditary, autosomal dominant disorder, the condition being referred to as hereditary multiple exostoses, multiple hereditary exostoses, or hereditary multiple osteochondromas [2, 4–7, 16]. In humans exhibiting this condition it has been shown that while the germline cells are heterozygous for the mutations of the *EXT* genes, the lesional cells are frequently found to be homozygous for the loss of *EXT* function [6], consistent with the two-hit hypothesis of tumorigenesis [17]. As not all human patients with hereditary multiple exostoses display loss-of-function mutations in *EXT* genes, also other mechanisms causing the condition have been postulated [6].

Osteochondromatosis in the domestic cat markedly differs from that in humans, domestic dogs, and horses and is therefore considered non-analogous to the latter condition. Osteochondromatosis in the cat is non-hereditary and often involves flat rather than long bones [2, 7]. Moreover, the cortex of the underlying parent bone often remains intact, and there is no continuity between its marrow cavity and that of the exostosis [7]. Virus particles resembling feline leukemia virus and feline sarcoma virus have been observed in the cartilage of these osteochondromas; however, the significance of this finding is unclear [7].

A case of multiple osteochondromas of the ribs has been described in a domestic pig [8]. Another case, affecting the right femur, was reported from a captive rhesus macaque, *Macaca mulatta* [18]. Multiple lesions resembling osteochondromas, along with extensive soft tissue ossifications, were found in a stranded southern right whale, *Eubalaena australis* [19]. Multiple exostoses assumed to be of a hereditary nature have also been reported in the Oligocene canid genus *Hesperocyon* [20].

In deer, previously only a few solitary osteochondromas have been reported in the scientific literature [21–23]. This paper describes, for the first time, a case of multiple osteochondromas in a member of the family Cervidae.

## Materials and methods

In September 2013, the antlered cranium of a white-tailed buck (*Odocoileus virginianus*) was found by a hunter in Morgan County, Georgia, USA (33°34’59.9”N, 83°26’23.4”W). Only a few postcranial bones were present at the site, indicating that predators and/or scavengers had dispersed the majority of the skeleton. The cause of death of the animal could not be established. Only the cranium was collected and submitted for study.

The antlered cranium was photographed with a digital camera (Canon EOS300D), and scanned by computed tomography (CT) in a Phillips Brilliance 64CT scanner (120 kV, 250 mAS, slice thickness 0.8 mm). For microscopic analysis, samples from two smaller exostoses and their parent bone were cut from the right and the left antler, respectively, and embedded in epoxy resin (Biodur E12, Biodur Products, Heidelberg, Germany). A further sample was taken from the exostosis and the underlying bone at the left zygomatic. The embedded specimens were bisected along the proximo-distal axis of the exostoses, and the cut surfaces of the blocks smoothed and polished as described previously [24].

The (uncoated) polished surfaces were viewed in a scanning electron microscope (SEM; Zeiss Evo Ma 15) operated in low pressure mode at 20 kV accelerating voltage, using a backscattered electron (BSE) detector. Variation in the intensity of the BSE signal caused by differences in the degree of mineralization is reflected by gray-level variation in the BSE images of the polished sections, with brighter gray levels corresponding to increased signal intensities and thus higher degrees of mineralization [25]. For better visualization, the gray-scale images obtained in the SEM were converted to pseudo-color images (mineralization maps) using the 16-colors lookup table of the ImageJ software package (NIH, Bethesda, USA). For that, the 256 gray levels from black (0) to peak white (255) were converted to 16 bands of equal width, each represented by a different color.

Following BSE imaging, the blocks were mounted with their polished sides down on microscopic slides, and ground sections of the specimens were produced as described previously [24]. The cover-slipped sections were then viewed in a Zeiss Axioskop 2 Plus microscope under plain and linearly polarized light. In the latter case, a full wave retardation plate (first order red plate) was inserted between specimen and analyzer.

## Results

**Fig 1:**
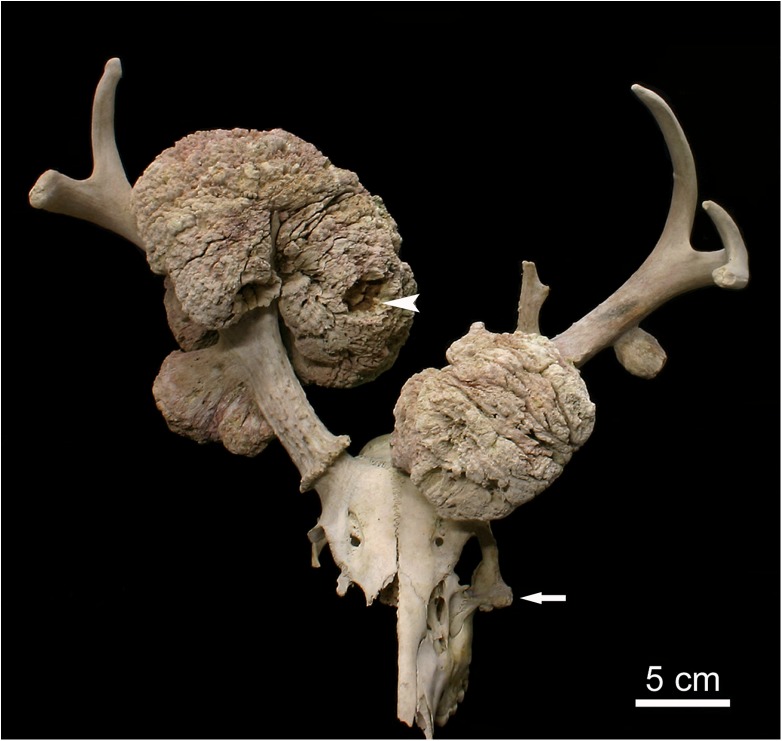
Antlered cranium of the white-tailed buck (*Odocoileus virginianus*), rostro-dorsal view. The antlers exhibit several pedunculated exostoses of different size. The surface of these exostoses is rugose and characterized by crevices and some deep crater-like depressions (arrowhead). Arrow: small, sessile exostosis on the left zygomatic bone.

The cranium was incomplete, with loss of the facial bones of the right side and of the left incisive bone, and devoid of soft tissue remains (Fig 1). The cranial bones and antlers were considerably weathered and sun-bleached, suggesting that they had been exposed to the environment for presumably a year or more. The left maxillary row of cheek teeth was complete and showed moderate wear (with exposure of dentin) on all permanent premolars (P^2-4^) and molars (M^1-3^), indicating a young adult age at death of the individual.

**Fig 2:**
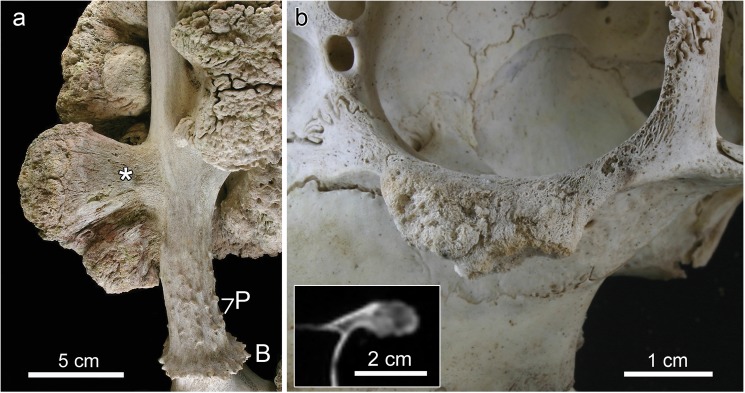
**Close-up views of exostoses arising from the right antler (a) and the left zygomatic bone (b) of the white-tailed buck (*Odocoileus virginianus*).** (**a**) Rostral view of the right antler showing the smaller bilobular exostosis and portions of the more distally located larger one. Note the smooth surface of the stalk (asterisk) and the rugose/nodular surface of the peripheral portion of the mass. B: burr at antler base; P: small protuberances (‘pearls’) of the antler surface. (**b**) Lateral view of the left zygomatic bone with broad-based exostosis. The insert shows a CT image demonstrating continuity of spongiosa and cortex of the exostosis with those of the parent bone.

Antler length, measured along the posterior curvature, was 32 cm on the right side and 31 cm on the left. The antlers proper were of species-specific shape, with the main beams directed backward in their basal portions and a forward curving of the upper segments (Fig 1). The tines (three on the right side, four on the left) pointed upward and, except for one, possessed rather blunt tips. As is typical for the species, the antlers exhibited small surface protuberances (‘pearls’) in their proximal portions. The more distal antler portions showed a rather smooth surface with shallow vascular grooves (Fig 2A). Both antlers possessed burrs (osteophytes arranged in a ring-like fashion) at their base. (Figs 1 and 2A). The morphological findings indicate that seasonal antler regeneration had been completed when the animal died. Whether or not the covering skin (velvet) had already been shed from the antlers at the time of death cannot be determined.

The antlers exhibited multiple exostoses of different size (Fig 1). The outer regions of the exophytic masses were partly stained brown-green, consistent with environmental weathering. The exostoses were pedunculated with a basal stalk arising from the antler surface and a broad tuberous distal region (Figs 1 and 2A). While the stalks (especially of the smaller lesions) exhibited a smooth surface (Fig 2A), the peripheral regions of the exostoses were rugose and nodular with numerous crevices and some crater-like depressions (Figs 1 and 2A). The peripheral portions of the two largest exostoses on the antlers were very brittle and porous.

**Fig 3:**
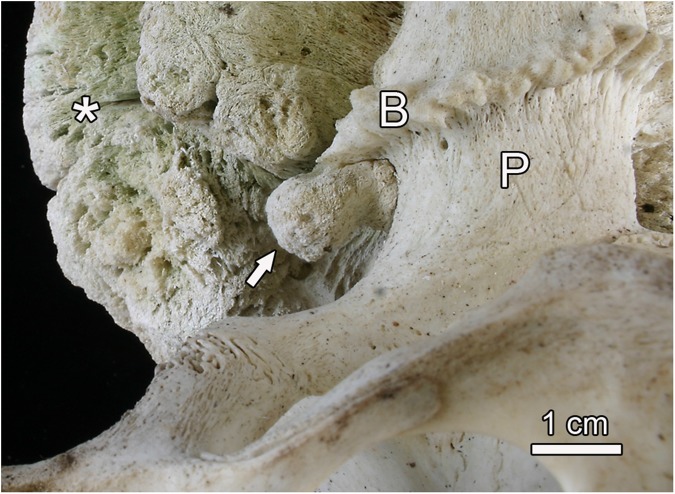
Small pedunculated exostosis (arrow) arising from the undersurface of the burr of the left antler of the white-tailed buck (*Odocoileus virginianus*). B: burr at the base of the left antler; P: pedicle; asterisk: portion of large exostosis arising from the left antler.

**Fig 4:**
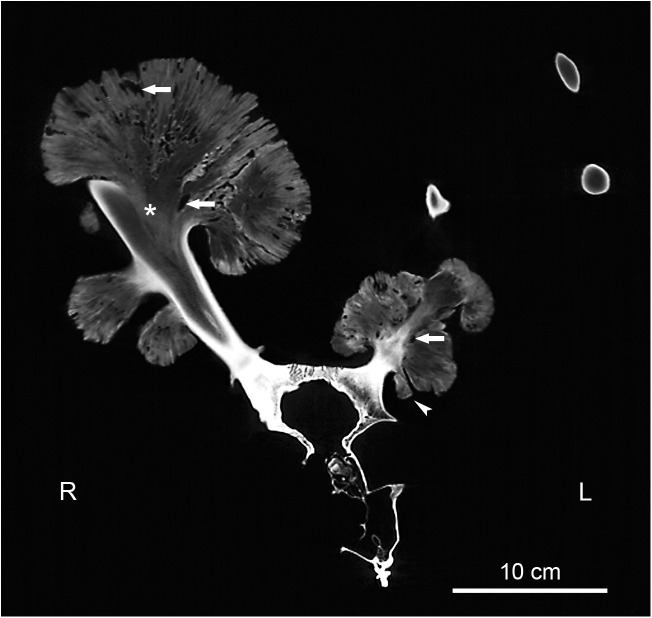
CT image showing the cranium and antlers of the white-tailed buck (*Odocoileus virginianus*). Note the normal composition of the antlers with a compact cortex and a cancellous core, as seen in the right antler and the two virtual cross sections of the distal part of the left antler in the upper right corner of the large image. Also note the continuity of the spongiosa (asterisk) and the cortex (present only in the basal stalk region) of the large exostosis from the right antler with the respective components of the parent bone. The seeming non-continuity of the cancellous bone between the antler and the smaller exostosis from the right antler as well as its seemingly bipartite structure is due to the fact that the slice is not located in the central axis of its stalk. Arrows: radiolucent areas within the outgrowths. R: right side; L: left side.

The right antler exhibited a large (length, measured perpendicular to the surface of the parent bone: 19.4 cm, maximum circumference: 55 cm) pedunculated exostosis that had partially overgrown the main beam and the lowermost tine (Fig 1). Further proximally, a smaller, bilobular exostosis (length: 7.1 cm) arose from the lateral antler surface (Fig 2A). The basal portion of the left antler showed a large (length. 8.3 cm, maximum circumference 37 cm) multilobular pedunculated exostosis (Fig 1). Further distally, a small pedunculated exostosis (length: 3.4 cm) arose from the lateral side of the left antler. A small exostosis (length: 1.1 cm) arose from the undersurface of the left burr (Figs 3 and 4). An additional exostosis (length: 1.2 cm) was present on the left zygomatic bone. The mass, which had grown from the infraorbital margin of the zygomatic bone, was broad-based and exhibited a rugose, knobby surface (Fig 2B).

CT imaging revealed that in unaffected regions away from the exostoses, the antlers showed the normal composition of a dense outer shell (cortex) and a cancellous core. Where exostoses had formed, the antler cortex was disrupted and the cancellous bone of the antlers was continuous with that of the outgrowths (Fig 4). CT imaging further demonstrated continuity between the antler cortex and the cortical bone that was in places present at the base of the exostoses (Fig 4). The overall radiodensity of the exostoses was low and corresponded to that of the antlers’ cancellous bone (Fig 4). CT imaging further revealed the occurrence of numerous radiolucent areas of different size and shape within the exostotic masses (Fig 4). Continuity of its spongiosa and cortex with the respective components of the parent bone was also observed in the exostosis arising from the left zygomatic (Fig 2B, insert).

**Fig 5:**
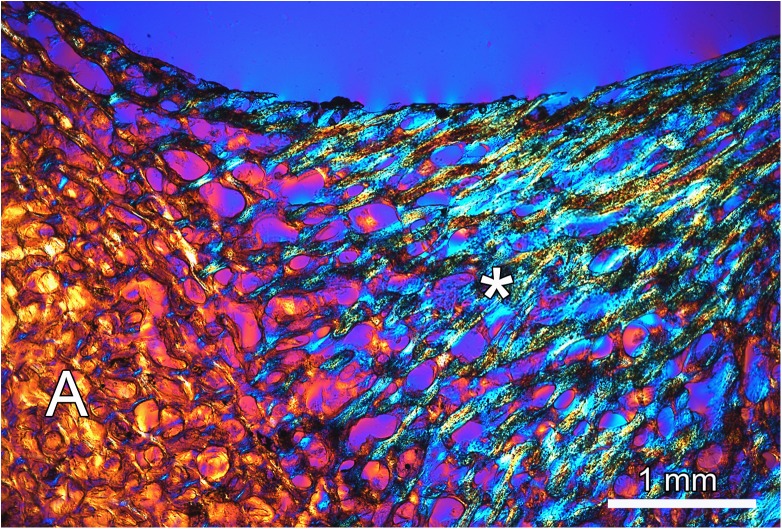
Micrograph of ground section, showing the basal region of the smaller exostosis (asterisk) on the left antler of the white-tailed buck (*Odocoileus virginianus*) (for location see Fig 1) and the underlying antler bone (A). Linearly polarized light plus full wave retardation plate. Note the change in collagen fiber orientation between the two portions as evidenced by the interference colors, and the continuity of the cancellous bone of the exostosis with that of the antler bone.

**Fig 6:**
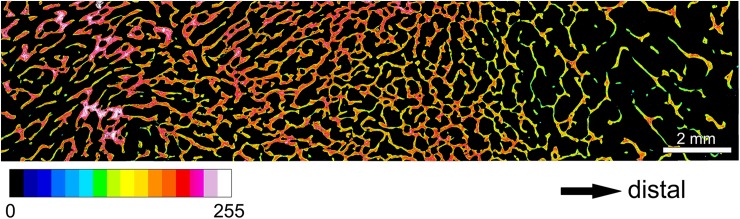
Stitched pseudo-color BSE image of the proximal portion (stalk region) of the smaller exostosis arising from the right antler of the white-tailed buck (*Odocoileus virginianus*). For location see Fig 2A. The gray levels from black (0) to peak white (255) obtained in the SEM were sequentially combined into16 bands of equal width, each represented by a different color (see inserted color bar). Higher values indicate higher degrees of mineralization. Note increasing tissue porosity and decreasing degree of mineralization of trabeculae in distal direction.

**Fig 7:**
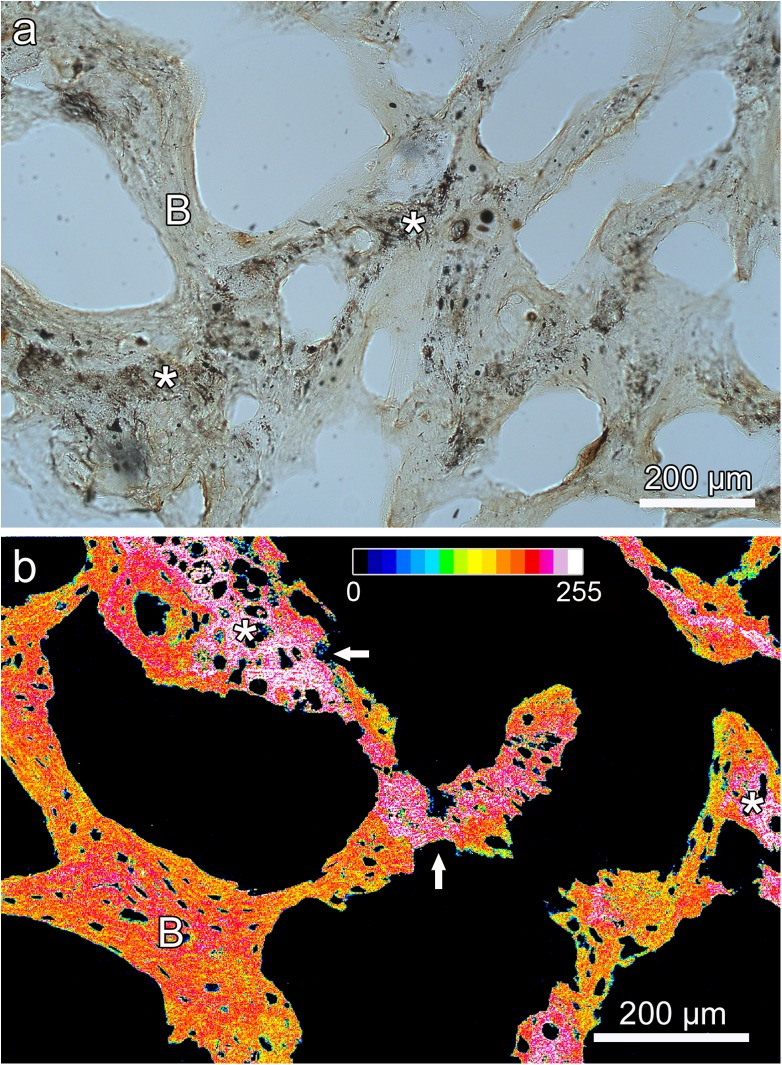
Images showing mineralized cartilage (asterisks) and bone (B) in the exostoses on the left zygomatic bone and right antler of the white-tailed buck (*Odocoileus virginianus*). (**a**) Micrograph of ground section, showing the central portion of the exostosis arising from the left zygomatic bone; plain transmitted light. (**b**) Pseudo-color BSE image of the tip region of the smaller exostosis arising from the right antler (depicted in Fig 2A). The gray levels from black (0) to peak white (255) obtained in the SEM were sequentially combined into16 bands of equal width, each represented by a different color (see inserted color bar). Higher values indicate higher degrees of mineralization. The mineralized cartilage shows a higher degree of mineralization than the adjacent bone. Note also the larger size, more variable shape and more irregular arrangement of the chondrocyte lacunae compared to the osteocyte lacunae. Arrows: resorption pits in mineralized cartilage.

When viewing the ground sections under polarized light, the trabeculae of the antler spongiosa and those forming the base of the exostoses could be easily distinguished due to their different collagen fiber orientation (Fig 5). At the base of the lesions, the trabeculae were preferentially oriented radially, indicating rapid growth of the exostoses. Microscopic analysis confirmed the continuity between the cancellous bone of the exostosis and that of the underlying antler (Fig 5). The stalks of the pedunculated exostoses consisted of cancellous woven bone of regular appearance, whose trabeculae became more loosely arranged, slender, and less mineralized in distal direction (Fig 6). Furthermore, the trabeculae were no longer preferentially oriented in the long axis of the exostoses, but arranged in a more reticular fashion. Towards the periphery of the exostoses, increasing amounts of mineralized cartilage were present (Fig 7). This mineralized cartilage often formed the more central portions of the trabeculae ((Fig 7A) and was typically more mineralized than the surrounding bone (Fig 7B). The mineralized cartilage frequently exhibited scalloped surfaces indicative of resorption pits (Fig 7B). Chondrocyte lacunae within the mineralized cartilage were of different shapes and rather irregular orientation (Fig 7B). The histological structure of the mass arising from the zygomatic bone corresponded to that of the antler exostoses.

## Discussion

The macroscopic, radiographic, and microscopic findings of the present study are consistent with a diagnosis of multiple osteochondromas affecting predominantly the antlers but also the left zygomatic bone of the white-tailed buck. The overall morphology of the exostoses closely resembles that of osteochondromas reported from humans and other mammals [3, 5–7]. In addition, the normal bone structure in the stalk regions of the exostoses and the gradual increase in the degree of bone mineralization towards the basal portions of the masses is more compatible with a benign than a malignant tumor. A further feature supporting the diagnosis as osteochondromas is the continuity of the cancellous and the compact cortical bone of the exostoses with the respective components of their parent bones that was demonstrated both by CT imaging and microscopic analysis.

In the present case, the cap of unmineralized hyaline cartilage characteristic of osteochondromas was lost in the process of decomposition, leaving only the mineralized portions of the exostoses. The presence/thickness of the cartilage caps could therefore not be assessed. The rather irregular orientation of the chondrocyte lacunae in the mineralized cartilage, in combination with the huge size of some of the exostoses, might be taken as an indication of malignancy, suggesting transformation of the osteochondroma into a secondary chondrosarcoma late during growth. However, considering that antler growth is the most rapid bone formative process known in mammals [26], the large size of some of the antler exostoses is not regarded a sufficient feature for diagnosing malignancy. Rather, the huge size of the lesions is considered to reflect the particular growth intensity of antlers in general. This view is supported by the much smaller size of the exostosis present on the left zygomatic bone of the buck.

Given the seasonal nature of antler growth, it can be concluded that the antler exostoses had developed within a period of only three to four months or even less. Considering that the buck’s antlers were regenerated ones, it would be interesting to know whether osteochondroma formation had also occurred in previously grown antlers. Unfortunately, no information on this is available.

The porous and brittle nature of the exostoses is consistent with postmortem degradation, and it is assumed that some erosion at their surface had occurred during and after skeletonization. The deep crater-like depressions in the surface of the larger exostoses probably represent areas formerly occupied by unmineralized cartilage that was lost during decomposition. The radiolucent areas present deeper within the tumors are likewise interpreted as indicative of former islands of unmineralized cartilage. Such foci of unmineralized cartilage within the cancellous portion of osteochondromas have been observed in humans [3, 6] and domestic animals [2].

Osteochondromas must be distinguished from peripheral osteosarcomas arising from undifferentiated cells of the outer fibrous layer (parosteal osteosarcoma) or the inner cambium layer of the periosteum (periosteal osteosarcoma) [2, 3, 6, 7]. Parosteal osteosarcomas are generally more radiodense than osteochondromas [6]. Moreover, contrary to osteochondromas, they typically show an intact cortex of the underlying bone [3, 6, 7]. In periosteal osteosarcomas, there may be some erosion of the outer cortex of the underlying bone, but the medullary cavity is also usually not invaded [6]. Parosteal and periosteal osteosarcoma were therefore excluded in the differential diagnosis. The continuity of their cancellous and compact bone with the respective components of the underlying parent bone distinguishes osteochondromas also from different forms of benign periosteal reactive lesions, including bizarre parosteal osteochondromatous proliferations [5, 6, 27], which were therefore also excluded.

In the case of antlers, osteochondromas must be differentiated from the pathological outgrowths, more recently referred to as antleromas [26, 28–30], that develop as a consequence of insufficient testosterone production. Experimentally, antleroma formation was induced by castration of male deer after completion of pedicle growth [28, 31, 32]. If castration occurs while a deer is carrying ‘hard’ antlers, i.e., antlers composed of bare bone devoid of velvet, the drop in testosterone causes premature antler casting, followed by the growth of a new set of antlers. The regenerated antlers do not undergo full maturation and remain permanently velvet-covered and viable. The latter also occurs when castration is performed during antler growth. Antleromas thus reflect sustained antler growth uncoupled from normal morphogenetic regulation [29, 30], with their shape, growth intensity and tissue composition differing both among and within species [26, 28–32]. Thus, e.g. for fallow deer (*Dama dama*) it was initially reported that antleromas consist of collagen masses with few interspersed fibroblasts and no bone [28], suggesting a dermal origin of the structures. A later study, however, demonstrated additional formation of bony outgrowths by periosteal intramembranous ossification, i.e., a case of hyperplastic bone growth, in antlers of castrated fallow bucks [32].

It is mostly agreed that, given their typical lack of invasive growth and metastasis, antleromas may be classified as benign tumors [28, 29, 32]. Recently, Munk et al. [30] reported a case of tumor growth on the antlers of a free-ranging white-tailed buck, in which the tumorous masses had displaced or replaced portions of the cranial bones. Despite their invasive growth, the tumors were classified as a case of bilateral antleroma, as they had maintained the basic histological structure of growing antlers [30]. However, given their aggressive presentation, the possibility remains that these tumors were of a malignant nature.

The antlers of castrated or hypogonadic white-tailed bucks are typically malformed and do not show a species-specific shape [26, 31]. In the case described by us, the exostoses had developed from the surface of otherwise normally shaped antlers. That the exostoses reported here constitute osteochondromas rather than antleromas is also demonstrated by the fact that the lesions are not confined to the antlers but that the zygomatic bone was also affected.

To our knowledge, the case presented here is the first instance of multiple osteochondromas described in a deer species. Thus far, a single case of solitary osteochondroma (maximum circumference 41 cm) of the left antler from a yearling fallow buck has been reported [22]. Two cases of solitary osteochondroma affecting, respectively, a cervical vertebra [21] and the frontal bone [22] were reported in roe deer (*Capreolus capreolus*). A huge tumor weighing 4.8 kg that had developed from the frontal bone of a female white-tailed deer was likewise diagnosed as an osteochondroma [23]. That osteochondromas in deer have thus far not been reported in limb bones, the common sites of osteochondroma formation in humans and most other mammals, is probably due to a sampling bias. While antlers receive considerable attention as trophies and deer skulls are regularly inspected for age estimation, less attention is normally paid to other regions of the skeleton.

The precise cellular origin of osteochondromas is unresolved [3, 4, 9] and may possibly vary between exostoses formed at different locations. Exostoses formed in the vicinity of a growth plate could originate from growth-plate chondrocytes, progenitor cells in the perichondrium, or cells located in the groove of Ranvier, a specialized perichondrial region near the epiphysis that may contain a stem cell niche [9]. There is evidence that, independent of their origin, the first detectable ectopic cells express early chondrocyte markers [9]. This suggest that osteochondroma formation is initiated in cells of the chondrocytic lineage.

Development of osteochondromas in long bones at sites distant to epiphyses and rarely even in membrane bones indicates that formation of these tumors can also occur unrelated to growth plates. It is therefore of interest that previously also other cellular origins of osteochondromas were discussed. Some authors suggested that osteochondromas arise from abnormal cartilage formation in the cambium layer of the periosteum [1], while others emphasized the occurrence of exostoses at entheseal sites, and considered osteochondromas to represent tumors of the precartilaginous connective tissue present in these locations [33].

Endochondral ossification is the dominant mode of bone formation in antlers [34–36]. However, in contrast to limb bones, a growth plate is missing in antlers. Antler elongation is driven by the proliferation of mesenchymal tissue located at the tips of the main beams and tines. Proximally, the mesenchymal cells sequentially differentiate into chondroblasts and chondrocytes. Subsequently, the chondrocytes undergo hypertrophy and the cartilage matrix is mineralized, followed by replacement of the mineralized cartilage by bone. Due to the short life span of antlers and the related virtual lack of remodeling of antler bone [24, 37], some mineralized cartilage remnants can persist within the bony trabeculae of mature antlers [24, 38, 39]. Antlers also show a limited growth in thickness by lateral apposition of bone, initially from the perichondrium and, after formation of an initial bone sleeve, the periosteum [35, 36].

As antlers lack both a growth plate and entheses, the cellular origin of osteochondromas in antlers must be sought elsewhere. Likely candidates are cells of the chondrocytic lineage located in the chondrogenic growth regions or progenitor cells in the perichondrium and periosteum at the flanks of the growing antlers. Chondrocytic differentiation of periosteally derived cells is also assumed in the case of the osteochondroma growing from the zygomatic bone of the white-tailed buck.

Considering that antlers are regularly collected as trophies and that large numbers of them are critically inspected by hunters each year, the rarity of reported antler tumors other than antleromas (with their specific hormonal cause) is striking and calls for an explanation [29]. A detailed discussion of this topic is beyond the scope of this contribution, but it may be noted that antlers share this apparent resistance to tumor formation with other structures capable of epimorphic regeneration [40]. This makes antlers highly interesting subjects for studying the action of tumor suppressor genes during rapid tissue growth and the effects of targeted disruption of these genes, an opportunity that has not been exploited so far.

In conclusion, we present a case of multiple exophytic masses arising from the antlers and the left zygomatic bone of a male white-tailed deer. Based on the available macroscopic, histological and radiographic evidence, the condition is diagnosed as a case of multiple osteochondromas. This is the first case of osteochondromatosis in deer reported in the scientific literature. Previously, only a single case of solitary osteochondroma on an antler has been reported, and antler osteochondromas must therefore be considered very rare tumors.
